# Prevalence of alopecia areata in Japan: Estimates from a nationally representative sample

**DOI:** 10.1111/1346-8138.16606

**Published:** 2022-11-22

**Authors:** Toshihiko Aranishi, Taisuke Ito, Masahiro Fukuyama, Yoshitaka Isaka, DeMauri S. Mackie, Kristen King‐Concialdi, Steven S. Senglaub, Dena H. Jaffe, Yutaka Shimomura, Manabu Ohyama

**Affiliations:** ^1^ Japan Drug Development and Medical Affairs Eli Lilly Japan K.K. Kobe Japan; ^2^ Department of Dermatology Hamamatsu University School of Medicine Hamamatsu Japan; ^3^ Department of Dermatology Kyorin University Faculty of Medicine Tokyo Japan; ^4^ Cerner Enviza Missouri North Kansas City USA; ^5^ Department of Dermatology Yamaguchi University Graduate School of Medicine Ube Japan

**Keywords:** alopecia areata, epidemiology, health‐related quality of life, Japan, prevalence

## Abstract

Data on the prevalence of alopecia areata (AA) in Japan is limited and the epidemiology of the disease there is not well understood; therefore, it is critical to examine the prevalence and severity of AA in Japan to inform the need for future treatments and research. A cross‐sectional, web‐based survey was conducted in Japan from January through March 2021. A total of 45 006 participants were identified through general population survey panels and asked about their experience with AA and hair loss. The Alopecia Assessment Tool and the Scalp Hair Assessment PRO^TM^ were adopted to screen for history of AA and assess disease severity, respectively. Eligible participants submitted photos of their scalp, which were reviewed by three board‐certified dermatologists to evaluate the presence and severity of AA. Prevalence and severity estimates were calculated using participants' self‐reported data and verified through the dermatologists' assessments. The participant‐reported point prevalence of AA was 2.18%. The adjusted point prevalence following physician adjudication using participant‐submitted photos was 1.45%. Topical corticosteroids were the most commonly used treatments, with 34.6% of participants diagnosed with AA reported having ever used them. Participants also reported negative impacts on their mood (70.2%), self‐esteem (55.8%), and social interactions (48.9%). Despite the social and emotional impact of hair loss, more than one third of those reporting a physician diagnosis of AA were not currently seeking treatment. The current study identified an estimated prevalence of AA in Japan between 1.45% and 2.18% based on the survey results and physician‐adjudication of those findings. Considering the impactful psychological burden of AA, the survey results showing that 38.90% of surveyed patients do not currently seek treatment may indicate an unmet need for remedies.

## 1 INTRODUCTION

Alopecia areata (AA) is an autoimmune skin disease that causes hair loss on the scalp, face, and other areas of the body.^1^ While AA is categorized as a type of nonscarring alopecia, without damage to the hair follicle stem cells, it can lead to persistent hair loss. Hair loss typically presents as patchy bald lesions on the scalp, beard, eyebrows, eyelashes, and other parts of the body and can progress to total scalp hair loss and/or eventually total body hair loss.^2^ AA has a variable course that may follow a relapsing and remitting pattern; speed of progression is often unpredictable and hair regrowth may occur in some areas while being irreversibly lost in others.^2^ Other types of alopecia include totalis, universalis, meandering or ophiasis, sisaipho, reticulate, incognita, and canities subita.^3^

The experience of hair loss with AA has been shown to be associated with significant impacts on patients' health‐related quality of life, including worsened emotional and mental health and vitality as disease severity worsens.^3^, ^4^ Persons with AA are at higher risk for depression and anxiety relative to persons without AA.^5^ Many also experience impaired social functioning as a result of their disease,^6^ an area of disease burden commonly overlooked. For instance, one prior study found that patients with AA experienced clinically significant levels of social anxiety, anxiety, and depression,^7^ and those who reported concerns about their decision whether to wear a wig reported even higher levels of impact. Thus, the impact on patients' health‐related quality of life is far‐reaching and when combined with these outcomes suggests a high burden of disease for patients with AA.

The worldwide lifetime incidence rate for AA has been reported to be approximately 2%^5^, ^6^, ^7^, ^8^; however, limited literature exists examining the prevalence of AA in Japan. For instance, a multicenter hospital‐based study among 170 clinics in Japan estimated the prevalence of AA in Japan at 2.5% among patients presenting for dermatological problems.^9^ While these data provide an approximation of the prevalence of AA in Japan, it is not a representation of AA among the general population; therefore, a general population‐based study is needed to further define the epidemiology of AA in Japan.

The Japanese Dermatological Association's guideline published in 2017 recommends a list of treatments, including corticosteroids (topical and intralesional) and topical therapies other than corticosteroids, represented by contact immunotherapy.^10^ However, some patients who use these treatments report limited efficacy and unfavorable adverse events.^11^ Given the level of burden that patients with AA experience, as well as the limited data on the number of people in Japan with AA, it is critical to examine the prevalence and severity of AA in Japan to inform the need for future treatments and research.

To address this gap in prior works, the present study aimed to determine the point prevalence (participant‐reported and physician‐adjusted) and lifetime prevalence (participant‐reported) of AA in a representative sample of the Japanese general adult population. Additionally, the study aimed to characterize disease history and treatment patterns for those reporting a diagnosis of AA within the Alopecia Areata Assessment Tool (ALTO).

## 2 METHODS

### 2.1 Measures

#### 2.1.1 Alopecia Assessment Tool

The ALTO is a diagnostic tool used to help discern AA from other types of hair loss.^12^ The tool contains eight closed‐ended questions, seven text‐based and one image‐based, which ask about an AA diagnosis from a dermatologist or other healthcare provider and the types and extent of hair loss experienced. The tool was designed with the intention of characterizing AA for large, epidemiological studies. For details on ALTO scoring, see Figure S1. There are nine scoring algorithms that capture the various ways respondents may answer and still be considered to have AA, all with differing levels of sensitivity and specificity (Table S1). A sensitivity analysis was conducted for the nine ALTO algorithms comparing the results of the physician evaluation of photographs and the patient‐reported outcomes to determine these metrics for each algorithm. It is important to note that the ALTO screens for a history of AA, and those who screen positive for AA may not currently be experiencing hair loss.

#### 2.1.2 The Scalp Hair Assessment PRO^TM^

The Scalp Hair Assessment PRO (SHA‐PRO^TM^) was developed as a patient‐reported severity assessment of current scalp hair loss.^13^ Participants were asked to estimate the total area of their scalp that is currently missing hair. Response options included no missing hair (0% of scalp), a limited area (1%–20%), a moderate area (21%–49%), a large area (50%–94%), and nearly all or all (95%–100%).

#### 2.1.3 Severity of Alopecia Tool

The Severity of Alopecia Tool (SALT) is a commonly used measure for determining the severity of AA based on the percentage of scalp hair loss in four main quadrants: the left, right, top, and back of the scalp.^14^ Each quadrant is evaluated independently, and scores are weighted based on the amount of surface area they cover (left/right = 18%; top = 40%; back = 24%). These scores are summed for a total percentage of hair loss, which is referred to as the SALT score. In this study, physicians independently determined a SALT score for each participant and scores were averaged. Individual participant scores were categorized as described in the Analysis section.

### 2.2 Study design

To understand the prevalence and severity of AA in Japan, a cross‐sectional study was conducted with adults (20 years or older) in Japan between January and March 2021. A nationally representative sample was targeted to reflect 2019 census estimates using age, sex, and geographic region. Survey participants were identified utilizing household survey panels and were asked to complete a web‐based survey on their health and hair loss experiences. Those meeting specific eligibility criteria were invited to contribute photos of their scalp for physician evaluation. The study obtained approval via the ethics committee at Saga Memorial Hospital (approved as of January 12, 2021).

### 2.3 Survey design

The participant survey incorporated questions on demographics, health conditions, outcome assessment measures, personal and familial hair loss experience, and treatment history. The survey was divided into two main sections: (1) a “general population” section, which was asked of all participants and included demographics, health conditions, ALTO, and the SHA‐PRO^TM^; and (2) a “hair loss or alopecia” section, which was asked of those meeting specific criteria outlined below and asked detailed questions regarding participants' hair loss or alopecia experience including impact of the disease, personal and familial history, and treatment history.

### 2.4 Participant inclusion

Participants were eligible to take part in the survey if they were an adult (aged ≥20 years), resided in Japan at the time of the survey, were able to read and complete a web‐based survey in Japanese, and provided informed consent. All participants, regardless of hair loss experience, were asked to complete the demographic questions, ALTO, and SHA‐PRO^TM^.

Participants who self‐reported a diagnosis of AA by a dermatologist or healthcare provider, scored positively on any of the nine ALTO algorithms (Table S1), or answered questions that characterized their AA as the meandering phenotype were asked to continue to the latter sections of the survey, which focused specifically on hair loss experience, including impacts and history of the disease and treatment. To assist in capturing participants with a meandering phenotype who would have otherwise screened out based on the ALTO criteria, a set of questions were developed based on Pratt et al. (2017)^2^ and Darwin et al. (2018).^15^ Questions focused on the type of hair loss (small or large round patches of hair loss or multiple patches of hair loss that join together) and location (hair line is receding and hair loss is on the back of scalp, sides of scalp, or neck) to determine whether the participant met the meandering criteria.

Participants who completed the full survey were then eligible for the photo submission if they were:

1. positive for AA based on the ALTO^12^ algorithm 5 (ALTO5; specifically, those who responded affirmatively to ALTO question 3 and any one of the following: 3a, 3b, 4, or 5) (Table S1),
2. those who responded “yes” to ALTO questions 1 or 2 (ever been diagnosed with AA), or
3. those who met the inclusion criteria for the meandering phenotype of AA as described above.

To ensure hair loss would be visible in the photos, participants who met these criteria were only eligible to submit photos if they reported missing any amount of hair currently (>0%) on the SHA‐PRO^TM^. Participants were excluded from the photo submission if they self‐reported: (1) a diagnosis of trichotillomania or telogen effluvium in the past 12 months; (2) hair loss in the past 12 months attributable to cancer treatment; (3) shaving or waxing an area affected by hair loss (reducing the ability to distinguish hair removal from AA); or (4) hair loss strictly caused by cuts, burns, or nicks from shaving. Criteria for the photo submission was designed to be as broad as possible to increase the number of photos submitted from patients with AA, therefore the least stringent ALTO algorithm (ALTO5) was used in these selection criteria.

Three board‐certified dermatologists in Japan independently evaluated the photo sets to diagnose alopecia and determine AA phenotype with final classifications determined by a majority rule. Physicians were also asked to estimate disease severity when reviewing the photo sets by using the SALT.^14^ Participant responses to specific questions including health history, ALTO, hair loss experience (past and current), and location of hair loss were provided to physicians to assist in the diagnosis and phenotype determination for each participant.

### 2.5 Analysis

Descriptive statistics including means and standard deviations (SDs) for continuous variables and frequencies and percentages for categorical variables were reported for all study measures.

#### 2.5.1 ALTO sensitivity and specificity calculation

To calculate sensitivity and specificity, the true‐positive (TP), false‐positive (FP), true‐negative (TN), and false‐negative (FN) rates were calculated for each algorithm. TP and TF were defined as those where the ALTO score matched the physician assessment, while those for whom the ALTO score and the physician assessment differed were defined as FP and FN, with the physician evaluation being the reference. Sensitivity was defined as (TP)/(TP + FN), and specificity was defined as (TN)/(TN + FP). An accuracy analysis was also conducted, which was defined as (TP + TN)/(total sample).

#### 2.5.2 Point prevalence calculation

The participant‐reported point prevalence rate of AA was calculated, along with 95% confidence interval (CI), as the proportion of participants who reported current hair loss (>0% hair loss on the SHA‐PRO^TM^) and screened positive for AA on ALTO5.

#### 2.5.3 Lifetime prevalence calculation

The lifetime prevalence of AA was calculated, along with 95% CI, as the proportion of participants who screened positive for AA according to the ALTO5 algorithm or screened positive for the meandering phenotype of AA.

#### 2.5.4 Participant‐reported severity

Participant‐reported severity was assessed using the SHA‐PRO^TM^ as described above. Participants were asked to inspect their hair using a mirror and assess the total amount of hair that was currently missing.

#### 2.5.5 Physician adjustments to point prevalence calculation

Physician adjustments were made to the reported unadjusted point prevalence calculation using the following formula:

$$\text{Unadjusted point prevalence}\times \left(\frac{\text{Number of physician determined}\;\mathrm{AA}\;\text{photos}}{\text{Number of physician evaluated photos}}\right).$$

#### 2.5.6 Physician‐adjudicated severity

Physician‐adjudicated severity was assessed using the SALT. Individual scores were averaged, and each participant was categorized as having mild (1%–20% hair loss), moderate (21%–49%), severe (50%–94%), or very severe (95%–100%) severity using categories developed in a study of dermatologists and patients with AA.^16^

## 3 RESULTS

### 3.1 Summary of recruitment and participant selection

**FIGURE 1:**
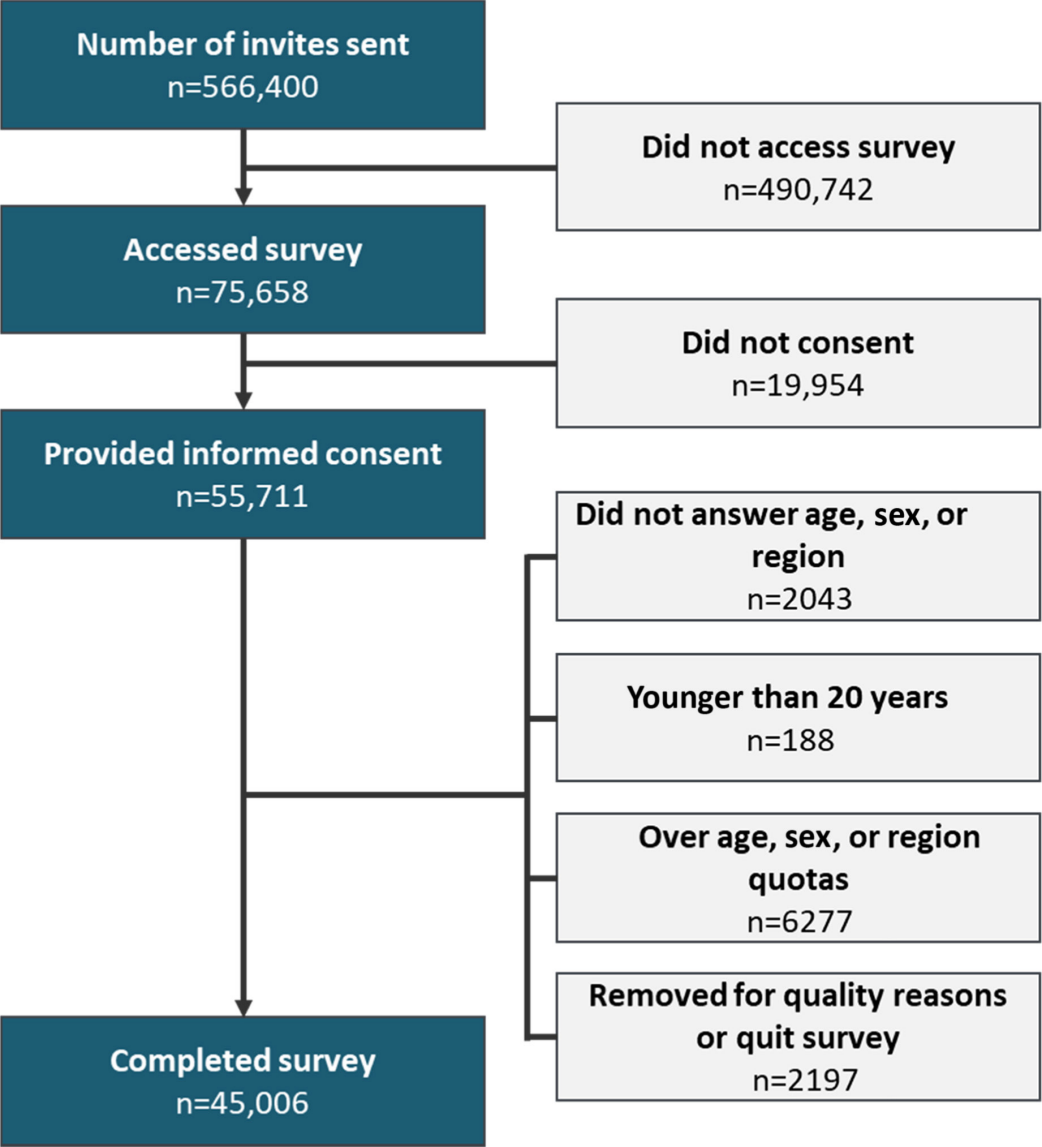
Survey response rate and reasons for exclusion or incompletion.

**FIGURE 2:**
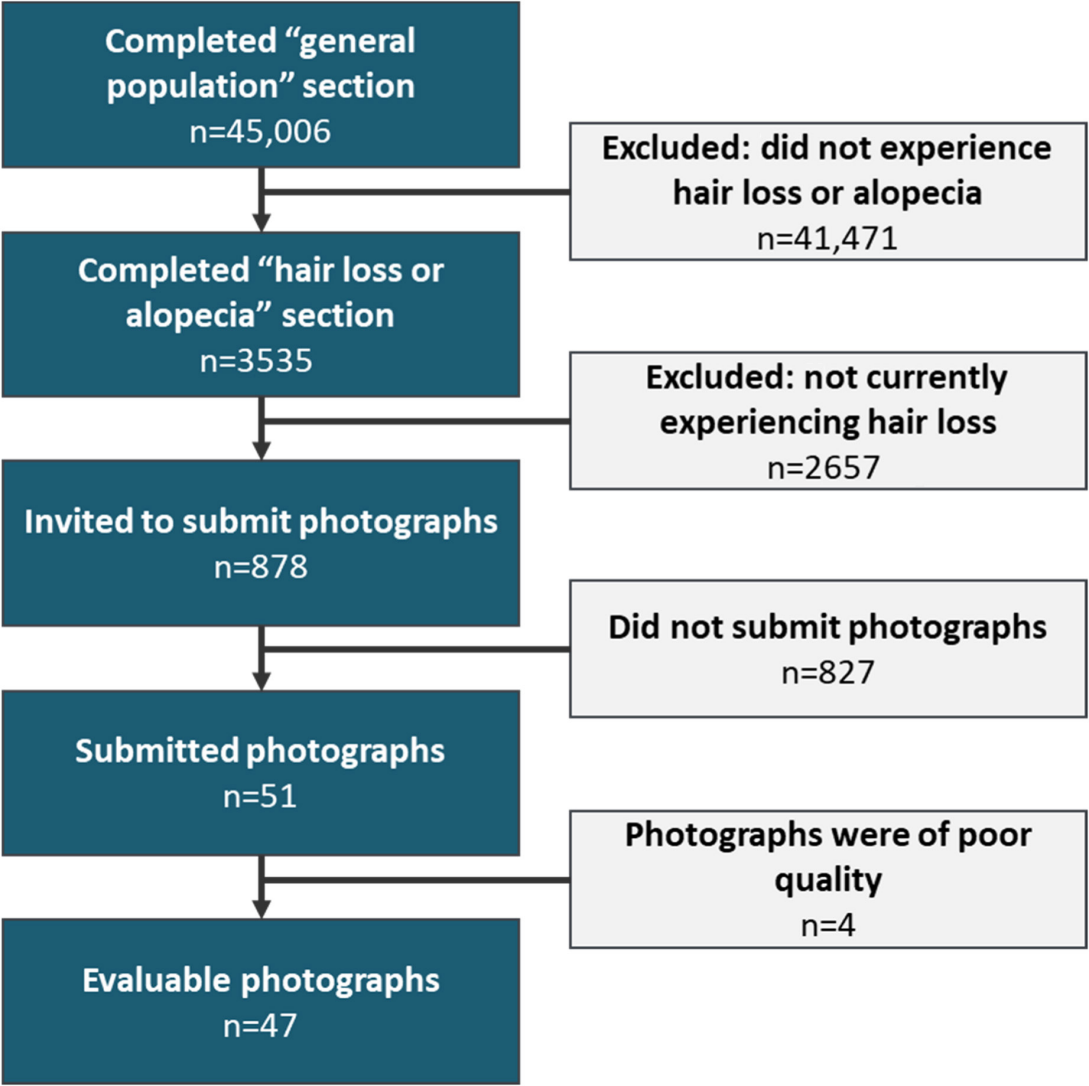
The respondent flow through the survey and reasons for exclusion of photo submissions.

Invitations to participate in the study were sent to 566 400 individuals enrolled in online research panels. Invitations for the survey were sent via email or were available on the participants' panel portal. The invitations were generic and only mentioned health generally without the mention of AA, hair loss, or other specific conditions. Of these, 75 658 individuals accessed the invitation and 55 711 provided informed consent to participant in the survey. A total of 2043 participants were excluded because they did not provide answers to the sex, age, or region questions, which were essential for targeting a nationally representative sample. Another 188 were excluded because they were under the age of majority (20 years). To ensure that the sample reflected the general population, a total of 6277 respondents completed the survey but were not included in the final analysis because they were over the age, sex, or region quotas set to be a representative sample. Finally, 2197 participants did not complete the full survey (voluntarily quit) or were removed for quality reasons. The final participant sample included 45 006 respondents. Of this sample, 3535 were eligible and completed the survey in its entirety, 878 were invited to submit photos, and 47 photo sets were evaluated. The survey response rate is outlined in Figure 1 and the respondent flow is outlined in Figure 2.

### 3.2 Sample characteristics

Participants in the overall sample had a mean age of 52.47 years (SD = 16.20 years) and 47.97% were men. A total of 46.74% had completed a 4‐year college/university degree or higher. Annual household income was well distributed, with 45.14% falling below ¥5 000 000 (≈$44 000) per year. Among those who scored positive on ALTO5, participants had a mean age of 51.71 years (SD = 14.99 years) and 43.58% were men. A total of 43.89% had completed a 4‐year college/university degree or higher. See Table 1 for participant characteristics for the overall sample (*n* = 45 006) and the ALTO5 population (*n* = 2584).

**TABLE 1:** Participant characteristics (*n* = 45 006)

	Overall population (*n* = 45 006)		ALTO5 population (*n* = 2584)	
Age				
Mean, SD	52.47	16.20	51.71	14.99
Median, range	53	20.00–120.00	51.50	20.00–87.00
	Number	Percentage	Number	Percentage
Sex				
Male	21 588	47.97	1126	43.58
Female	23 418	52.03	1458	56.42
Prefer not to answer	0	0.00	0	0.00
Education				
Less than high school	943	2.10	76	2.94
High school graduate or equivalent	13 107	29.12	766	29.64
Completed 2‐year professional, vocational, or technical school	9564	21.25	593	22.95
Completed 4‐year college/university (e.g., BA)	18 381	40.84	970	37.54
Completed graduate school (e.g., MS, PhD, MD)	2655	5.90	164	6.35
Prefer not to answer	356	0.79	15	0.58
Annual household income, ¥				
<1 000 000	3132	6.96	158	6.11
1 000 000 to 2 999 999	6281	13.96	366	14.16
3 000 000 to 4 999 999	10 901	24.22	620	23.99
5 000 000 to 7 999 999	10 339	22.97	665	25.74
8 000 000 to 9 999 999	4011	8.91	269	10.41
≥10 000 000	4215	9.37	264	10.22
Prefer not to answer	6127	13.61	242	9.37
Current employment				
Business owner/self‐employed	3477	7.73	238	9.21
Business professional	6312	14.02	401	15.52
Educator	1409	3.13	90	3.48
Government/civil services	1623	3.61	81	3.13
Homemaker	7381	16.40	389	15.05
Hospitality	5474	12.16	356	13.78
Retired	2239	4.97	111	4.3
Sales	3596	7.99	224	8.67
Student	1071	2.38	40	1.55
Not employed	6391	14.20	327	12.65
Long‐ or short‐term disability	215	0.48	21	0.81
Other	5366	11.92	289	11.18
Prefer not to answer	452	1.00	17	0.66

Abbreviations: ALTO5, Alopecia Areata Assessment Tool algorithm 5; SD, standard deviation.

### 3.3 ALTO sensitivity and specificity

Using the sensitivity and specificity analysis defined above, ALTO5 had a sensitivity of 93.3%, a specificity of 26.7%, and an accuracy of 71.1%. Based on the high levels of sensitivity and accuracy compared with the other algorithms, and based on previous literature,^10^ ALTO5 was selected for use in all further analyses.

### 3.4 Prevalence estimates (point, adjusted, and lifetime)

The participant‐reported point prevalence of AA was 2.18% (95% CI, 2.04%–2.32%), which was calculated as the proportion of participants who reported currently having hair loss (>0% hair loss on the SHA‐PRO^TM^) and screened positive for AA on ALTO5. After adjusting the point prevalence based on the physician evaluation of photos, as described above, the point prevalence of AA was 1.45% (95% CI, 1.37%–1.59%). Participant‐reported lifetime prevalence was 5.74% (95% CI, 5.53%–5.96%) as shown in Table 2.

**TABLE 2:** Prevalence estimates

Variables	Estimate (%)	95% CI
Participant‐reported severity prevalence among the general population (*n* = 45 006)		
Participant‐reported point prevalence	2.18	2.04–2.32
Physician‐adjusted point prevalence	1.45	1.34–1.56
Participant‐reported lifetime prevalence	5.74	5.53–5.96
Participant‐reported severity prevalence among participants with AA based on diagnosis (*n* = 1928)		
No missing hair (0% of scalp is missing hair; a full head of hair)	61.00	58.78–63.18
A limited area (1%–20% of scalp is missing hair)	29.30	27.28–31.39
A moderate area (21%–49% of scalp is missing hair)	6.17	5.14–7.34
A large area (50%–94% of scalp is missing hair)	1.87	1.31–2.58
Nearly all or all (95%–100% of scalp is missing hair)	1.66	1.14–2.34
Participant‐reported severity prevalence among AA participants based on full population (*n* = 45 005)		
No missing hair (0% of scalp is missing hair; a full head of hair)	71.13	70.70–71.54
A limited area (1%–20% of scalp is missing hair)	21.15	20.78–21.53
A moderate area (21%–49% of scalp is missing hair)	4.66	4.47–4.86
A large area (50%–94% of scalp is missing hair)	1.59	1.48–1.71
Nearly all or all (95%–100% of scalp is missing hair)	1.47	1.36–1.59
Participant‐reported severity prevalence among participants with AA based on ALTO5 (*n* = 2584)		
No missing hair (0% of scalp is missing hair; a full head of hair)	62.07	60.17–63.95
A limited area (1%–20% of scalp is missing hair)	28.91	27.17–30.70
A moderate area (21%–49% of scalp is missing hair)	5.80	4.93–6.78
A large area (50%–94% of scalp is missing hair)	1.90	1.41–2.50
Nearly all or all (95%–100% of scalp is missing hair)	1.32	0.91–1.83
Physician‐adjudicated severity of submitted photos using the SALT (*n* = 30)	Percentage	Number
1%–20% hair loss	53.33	16
21%–49% hair loss	10.00	3
50%–94% hair loss	0.00	0
95%–100% hair loss	0.00	0
Physicians disagree whether photos are evaluable, unable to calculate	10.00	3
Physicians agree that photos are unevaluable, unable to calculate	26.67	8

Abbreviations: AA, alopecia areata; ALTO5, Alopecia Areata Assessment Tool algorithm 5; CI, confidence interval; SALT, Severity of Alopecia Tool.

### 3.5 Participant‐reported severity

Among those who were positive on ALTO5 (*n* = 2584 with a past or current history of AA), 3.22% of respondents stated that they were missing more than 50% of their hair. Among those who reported a diagnosis of AA (*n* = 1928), 3.53% stated that they were currently missing more than 50% of their scalp hair. Among the full population (*n* = 45 006), 3.06% stated that they were currently missing more than 50% of their scalp hair (Table 2).

### 3.6 Physician‐adjudicated severity

Eligible participants were asked to submit photos of their scalp if they completed the survey in its entirety. Based on these criteria, 878 participants were invited to submit photos, 51 participants submitted photos, and 47 sets of photos were determined to be evaluable by the physician panel based on photo quality and the proportion of the scalp included in the photos.

Based on a majority rule of independent evaluations, 30 participants were determined to have probable or definite AA. Based on the SALT score, 16 participants (53.33%) were determined to have limited area of hair loss (1%–20% of their scalp), three participants (10.00%) were determined to have a moderate area of hair loss (21%–49% of their scalp), and the remainder were not able to be evaluated for severity because of poor photo quality (Table 2).

### 3.7 Impacts of hair loss

Analyses also examined the impact of hair loss on participants' emotional and social well‐being among those reporting a diagnosis of AA (*n* = 1928) (Table 3). Among this population, over two‐thirds (70.18%) reported that AA impacted their mood, over half (55.76%) reported AA having a negative impact on their self‐esteem or self‐confidence, and nearly half (48.91%) reported an impact on their social interactions.

**TABLE 3:** Impact of hair loss and disease history among physician‐diagnosed AA (*n* = 1928)

Variables		Percentage		Number	
Self‐esteem or self‐confidence		55.76		1075	
Overall mood		70.18		1353	
Social life or public interaction		48.91		943	
Personal or romantic relationships		38.38		740	
Basic activities of daily living		34.28		661	
Performance at school or work		23.65		456	
Relationships with colleagues or peers		27.44		529	
Other areas not listed		28.58		551	
**Variables**	**Number**	**Mean**	**SD**	**Median**	**Minimum–maximum**
Number of patches of hair loss	1713	1.94	6.44	1	1.00–99.00
Age at first hair loss, years	1717	30.03	14.91	28	1.00–85.00
Time since AA diagnosis, years	1928	20.69	16.43	18.08	0.08–72.58

Abbreviations: AA, alopecia areata; SD, standard deviation.

### 3.8 Disease history

Among those reporting a diagnosis of AA (*n* = 1928), participants reported first experiencing hair loss at a mean age of 30.03 years (SD = 14.91 years) and a mean time since initial diagnosis of 20.69 years (SD = 16.43 years). Participants also reported an average number of patches of hair loss of 1.94 (SD = 6.44) (Table 3).

### 3.9 Treatment history (ever and current)

Among those reporting a diagnosis of AA (*n* = 1928), 43.57% of participants reported that they had not ever been treated for their AA. The most commonly used treatments were topical corticosteroids, of which 8.87% of participants reported current use and 34.60% reported having ever used them. Vasodilators (e.g., minoxidil 5% [topical] and carpronium chloride hydrate [topical]) were also common, as 3.89% reported current use and 10.63% reported use at any time. Oral corticosteroids such as prednisolone were currently being used by 3.27% of participants and had ever been used by 7.37% of participants. Intralesional steroid injections (e.g., methylprednisolone and triamcinolone acetonide) were slightly less common, as they were currently being used by 1.66% of participants and had ever been used by 6.90% of participants. Last, 1.14% reported current use of phototherapy and 7.42% reported use of phototherapy at any time (Table 4).

**TABLE 4:** AA treatment history among participants reporting a diagnosis of AA (*n* = 1928)

Variables	Percentage	Number
AA treated at any time or currently	—	1928
Yes	56.43	1088
No	43.57	840

Treatments used	At any time		Current	
	Percentage	Number	Percentage	Number
Corticosteroids – topical ointment, cream, or foam (e.g., antebatea)	34.60	667	8.87	171
Corticosteroids – oral (pill) (e.g., prednisolone)	7.37	142	3.27	63
Corticosteroids – injection (e.g., methylprednisolone and triamcinolone acetonide)	6.90	133	1.66	32
Vasodilators (topical: minoxidil 5% and/or carpronium chloride hydrate)	10.63	205	3.89	75
Immunosuppressant (e.g., tacrolimus, methotrexate, and cyclosporine)	2.49	48	1.14	22
Immunomodulators (e.g., tofacitinib, ruxolitinib, and dupilumab)	0.88	17	0.36	7
Topical immunotherapy (e.g., squaric acid dibutylester)	2.28	44	0.52	10
Cepharanthine (oral)	2.96	57	0.99	19
Glycyron (oral)	1.61	31	0.67	13
Phototherapy	7.42	143	1.14	22
Liquid nitrogen therapy	4.46	86	0.41	8
Alternative medicines/home remedies (e.g. biotin, vitamins, essential oil, herbal treatments, and hair oil)	3.22	62	1.50	29
Other	4.30	83	0.52	10
I am not currently taking anything for my AA	38.90	750	—	—

Abbreviation: AA, alopecia areata.

## 4 DISCUSSION

The present study explored the epidemiology of AA in adults in Japan and showed a participant‐reported point prevalence of 2.18% and an adjusted point prevalence of 1.45%. In addition, considering the clear psychological burden of AA, the result of this study indicated that 43.57% of surveyed patients have never been treated before, which may suggest the presence of an unmet need for remedies. The current study reports the real‐world experiences of adults in Japan with hair loss and AA and describes their demographics, health, and treatment history, as well as participant‐reported estimates of disease severity. In summary, the current study represents an important contribution to the research literature on the prevalence of AA in Japan.

Prevalence estimates calculated in the present study are derived from a representative sample of the Japanese adult population and are supported by a physician‐adjusted point prevalence estimate, both designed to increase the accuracy and validity of the findings. The study design was based on previous studies in the United States^12^, ^17^ and included robust sampling techniques for determining point prevalence estimates. We note that the population of participants categorized as having AA in the current study had a higher mean age and a higher percentage of male participants than typically seen in AA populations, possibly owing to the inclusion of adult patients only. The observed prevalence estimates may also differ from those previously published for a variety of reasons. For example, prevalence rates are influenced by the study population. Thus, although our findings supplement prior work by Furue et al., which estimated a 2.5% prevalence rate of AA in Japan,^8^ higher rates in the latter were likely attributable to the study sample of patients who sought care for their hair loss compared with the current study population of all persons with AA. Additionally, while AA may be underdiagnosed in the general population because of its similarity to other forms of hair loss commonly linked to aging hair loss or androgenic alopecia,^16^ the present study utilized a validated AA screening tool to identify those without a formal diagnosis of AA but who still met criteria for the disease.

Approximately 75% of participants reported that hair loss resulted in negative effects on their quality of life, in particular with negative impacts on their self‐esteem, mood, and social interactions. These estimates are in line with previously published results.^3^, ^5^ This burden is compounded by our findings of the magnitude and extent to which those seek treatment. Specifically, slightly over half (56.4%) of participants who reported a diagnosis of AA had ever treated their condition, suggesting that nearly half of patients with AA may not seek or receive any treatment. There are several potential explanations for these findings: (1) persons with AA may experience mild symptoms and do not wish to treat their AA; (2) persons with AA are not seen by healthcare providers or are unaware of the latest or current treatments available; (3) persons with AA are unaware that treatment for hair loss may exist; (4) persons with severe AA may hesitate to show their scalp to anyone, even a physician, and may therefore not seek treatment; and (5) persons with AA may not access the healthcare system because of the COVID‐19 pandemic.^18^

Misdiagnosis and undertreatment of AA are also related to a societal attitude toward the disease. Historically, AA was erroneously thought to be a symptom of mental illness and it was unknown that AA is an autoimmune disease.^19^ This mischaracterization of AA was a factor in the delayed development of appropriate and effective treatments targeting the correct mechanism of action of AA. In these circumstances, patients may lose their motivation to find treatment, which may, in part, explain the low proportion of treated patients. The present study suggests that there is an opportunity for increased physician education, which may reduce the frequency of misdiagnosis and undertreatment of AA. Enhancing disease knowledge among physicians and patients may motivate patients with new AA symptom onset to access the healthcare system. Further, in the general population, familiarity with AA and its treatment may assist in targeting persons hesitant to seek care.

Although the present study provides critical prevalence estimates for AA in a nationally representative sample in Japan, there are some limitations that should be noted. The survey targeted the general population of Japan based on age, sex, and region. However, while the current study asked respondents to describe their AA history, including their AA history during childhood, it did not capture current experiences of pediatric AA. Therefore, any conclusions must be limited to the adult population. Also, while the survey was designed to include those with AA and not another form of alopecia or hair loss, participants with non‐AA or another form of hair loss may have been classified with AA. Overlap between androgenic alopecia and AA could be further explored in a post hoc analysis.

As with any self‐reported study, recall and self‐presentation biases may have occurred. As participants were remunerated, incentive‐based bias may also exist. Further biases may include a lack of representation caused by absence of internet or electronic device access or discomfort using such technology or survey platforms. To minimize bias, the survey was labeled as a general health survey and did not disclose the focus of AA until time of consent. Eligibility questions were worded in a nonsuggestive manner to reduce the opportunity for manipulation of the answers. Participants were required to answer each question before moving on and missing data were therefore nonexistent.

With regard to the COVID‐19 pandemic, the current survey was conducted during a state of emergency in Japan, which was declared in several prefectures and encouraged residents to stay home. This declaration as well as an overall cautiousness toward nonessential public or social interaction may have imposed limitations or caused behavior changes among persons with AA who typically seek treatment, such as limited access to stores and pharmacies that carry treatments for AA or a lessened interest in treating AA based on reduced social gathering.

Finally, the present study estimated that 100 sets of photos would be submitted based on the total sample size and prior submission rates in similar studies. While the photo submission criteria were designed to be as broad as possible, including those who reported a diagnosis of AA or who screened positive for AA in our survey algorithm, only 51 sets of photos were submitted. The small number of photos submitted and evaluated may limit the generalizability of the physician‐adjudicated results. The submission rate may have been limited by the participants' desire to maintain their privacy or the amount of effort required to submit photos. Since photos were self‐submitted, photo quality and photo angle were restricted by the skill and camera quality of the participants; in some cases, photos were unable to be evaluated because of the quality or perspective of the photo. It is also worth noting that assessments performed in the clinic may be more accurate than those conducted with photographs based on the ability to manipulate hair and view various angles of the scalp; however, this limitation was mitigated by asking participants to pin their hair to show areas of hair loss, if possible. There were no submitted photos that were determined through the SHA‐PRO^TM^ to have more than 50% hair loss, which prevented a physician‐adjusted analysis of severity. In addition, the sensitivity analyses conducted for the ALTO algorithms were limited by the number of photo sets submitted.

## 5 CONCLUSIONS

The current study reports a participant‐reported point prevalence of AA at 2.18% and a physician‐adjusted point prevalence of AA at 1.45%. Despite negative impacts on quality of life, including social and emotional well‐being, 43.6% of participants diagnosed with AA never sought treatment, indicating an unmet need for further treatments, advocacy, or education for AA in Japan.

## FUNDING INFORMATION

The current study was funded by Eli Lilly & Company.

## CONFLICT OF INTEREST

T.A. and Y.I. are employees of Eli Lilly Japan K.K. and shareholders of Eli Lilly and Company. T.I. receives advisory fees from Eli Lilly Japan K.K. and receives research grants for studies not related to this work from Pfizer Japan Inc., Maruho Co. M.F. serves as an investigator of this research project and received technical fees from Eli Lilly Japan K.K. Y.S. receives advisory fees from Eli Lilly Japan K.K. and Maruho Co. and receives research grants for studies not related to this work from Eli Lilly Japan K.K., Maruho Co., and Sun Pharma Japan Ltd. D.M., K.K.C., S.S., and D.J. are employees of Cerner Enviza, which was hired by Eli Lilly to conduct this study. M.O. receives advisory fees from Eli Lilly Japan K.K., Pfizer Japan Inc., Janssen Pharmaceutical KK., Taisho Pharmaceutical Co., and ROHTO Pharmaceutical Co. and receives lecture fees form Eli Lilly Japan K.K. and research grants for studies not related to this work from Maruho Co., Sun Pharma Japan Ltd., and Shiseido Co.

## Supporting information


Appendix S1

## References

[1] National Alopecia Areata Foundation (NAAF) . What you need to know about alopecia areata. 2021.

[2] Pratt CH , King LE , Messenger AG , Christiano AM , Sundberg JP . Alopecia areata. Nat Rev Dis Primers. 2017;3:17011. 28300084 10.1038/nrdp.2017.11 PMC5573125

[3] Shi Q , Duvic M , Osei JS , Hordinsky MK , Norris DA , Price VH , et al. Health‐Related Quality of Life (HRQoL) in alopecia areata patients‐a secondary analysis of the National Alopecia Areata Registry Data. J Investig Dermatol Symp Proc. 2013;16:S49–50. 10.1038/jidsymp.2013.18 24326555

[4] Rencz F , Gulacsi L , Péntek M , Wikonkal N , Baji P , Brodszky V . Alopecia areata and health‐related quality of life: a systematic review and meta‐analysis. Br J Dermatol. 2016;175:561–71. 26914830 10.1111/bjd.14497

[5] Fricke ACV , Miteva M . Epidemiology and burden of alopecia areata: a systematic review. Clin Cosmet Investig Dermatol. 2015;8:397. 10.2147/CCID.S53985 PMC4521674 26244028

[6] Mostaghimi A , Napatalung L , Sikirica V , Winnette R , Xenakis J , Zwillich SH , et al. Patient perspectives of the social, emotional and functional impact of alopecia areata: a systematic literature review. Dermatol Ther. 2021;11:867–83. 10.1007/s13555-021-00512-0 PMC8163950 33770385

[7] Montgomery K , White C , Thompson A . A mixed methods survey of social anxiety, anxiety, depression and wig use in alopecia. BMJ Open. 2017;7:e015468. 10.1136/bmjopen-2016-015468 PMC5566602 28473521

[8] Mirzoyev SA , Schrum AG , Davis MDP , Torgerson RR . Lifetime incidence risk of alopecia areata estimated at 2.1% by Rochester Epidemiology Project, 1990‐2009. J Invest Dermatol. 2014;134:1141–2. 24202232 10.1038/jid.2013.464 PMC3961558

[9] Furue M , Yamazaki S , Jimbow K , Tsuchida T , Amagai M , Tanaka T , et al. Prevalence of dermatological disorders in Japan: a nationwide, cross‐sectional, seasonal, multicenter, hospital‐based study. J Dermatol. 2011;38:310–20. 21426384 10.1111/j.1346-8138.2011.01209.x

[10] Tsuboi R , Manabe M , Amo Y . Japanese Dermatological Association alopecia areata clinical practice guidelines 2017. Jpn J Dermatol. 2017;127:2741–62.

[11] U.S. Food and Drug Administration . The voice of the patient: a series of reports from the U.S. Food and Drug Administration's (FDA's) Patient‐Focused Drug Development Initiative: Alopecia Areata. 2018.

[12] Li DG , Huang KP , Xia FD , Joyce C , Scott DA , Qureshi AA , et al. Development and pilot‐testing of the Alopecia Areata Assessment Tool (ALTO). PLoS One. 2018;13:e0196517. 29874239 10.1371/journal.pone.0196517 PMC5991373

[13] Wyrwich KW , Kitchen H , Knight S , Aldhouse NVJ , Macey J , Nunes FP , et al. Development of the Scalp Hair Assessment PRO™ measure for alopecia areata. Br J Dermatol. 2020;183:1065–72. 32163589 10.1111/bjd.19024 PMC7754291

[14] Renert‐Yuval Y , Guttman‐Yassky E . The changing landscape of alopecia areata: the therapeutic paradigm. Adv Ther. 2017;34:1594–609. 28646393 10.1007/s12325-017-0542-7 PMC5504208

[15] Darwin E , Hirt PA , Fertig R , Doliner B , Delcanto G , Jimenez JJ . Alopecia areata: review of epidemiology, clinical features, pathogenesis, and new treatment options. Int J Trichology. 2018;10:51–60. 29769777 10.4103/ijt.ijt_99_17 PMC5939003

[16] Wyrwich K , Kitchen H , Knight S , Aldhouse NVJ , Macey J , Nunes FP , et al. The Alopecia Areata Investigator Global Assessment scale: a measure for evaluating clinically meaningful success in clinical trials. Br J Dermatol. 2020;183:702–9. 31970750 10.1111/bjd.18883 PMC7586961

[17] Benigno M , Anastassopoulos KP , Mostaghimi A , Udall M , Daniel SR , Cappelleri JC , et al. A large cross‐sectional survey study of the prevalence of alopecia areata in the United States. Clin Cosmet Investig Dermatol. 2020;13:259–66. 10.2147/CCID.S245649 PMC7131990 32280257

[18] Jaffe DH , Lee L , Huynh S , Haskell TP . Health inequalities in the use of telehealth in the United States in the lens of COVID‐19. Popul Health Manag. 2020;23:368–77. 32816644 10.1089/pop.2020.0186

[19] Reinhold M . Relationship of stress to the development of symptoms in alopecia areata and chronic urticaria. Br Med J. 1960;1:846–9. 14437190 10.1136/bmj.1.5176.846 PMC1967069

