# Supplementary material for: Prevalence of alopecia areata in Japan: Estimates from a nationally representative sample
Source: J Dermatol. 2022 Nov 22;50(1):26–36. doi: 10.1111/1346-8138.16606 (PMC10100223; doi:10.1111/1346-8138.16606)
Supplement: Supplementary file 1 — Appendix S1 [file JDE-50-26-s001.docx]

**Supplementary Table 1: ALTO sensitivity and Specificity Analysis**

| **Algorithm** | ***n positive*** | **Sensitivity** | **Specificity** | **Positive Predictive Value** | **Negative Predictive Value** | **Accuracy** | **Error rate** |
| --- | --- | --- | --- | --- | --- | --- | --- |
| ***Estimates (95% confidence intervals)*** | | | | | | | |
| ALTO 1 | 20 | 56.7%  (38.9%-74.4%) | 80.0%  (59.8%-100.0%) | 85.0%  (69.4%-100.0%) | 48.0%  (28.4%-67.6%) | 64.4% | 35.6% |
| ALTO 2 | 20 | 56.7%  (38.9%-74.4%) | 80.0%  (59.8%-100.0%) | 85.0%  (69.4%-100.0%) | 48.0%  (28.4%-67.6%) | 64.4% | 35.6% |
| ALTO 3 | 12 | 40.0%  (22.5%-57.5%) | 100.0%  (100.0%-100.0%) | 100.0%  (100.0%-100.0%) | 45.5%  (28.4%-62.4%) | 60.0% | 40.0% |
| ALTO 4 | 21 | 56.7%  (38.9%-74.4%) | 73.3%  (51.0%-95.7%) | 81.0%  (64.2%-97.7%) | 45.8%  (25.9%-65.8%) | 62.2% | 37.8% |
| ALTO 5 | 39 | 93.3%  (84.4%-102.3%) | 26.7%  (4.3%-49.0%) | 71.8%  (57.7%-85.9%) | 66.7%  (28.9%-104.3%) | 71.1% | 28.9% |
| ALTO 6 | 39 | 93.3%  (84.4%-102.3%) | 26.7%  (4.3%-49.0%) | 71.8%  (57.7%-85.9%) | 66.7%  (28.9%-104.3%) | 71.1% | 28.9% |
| ALTO 7 | 20 | 56.7%  (38.9%-74.4%) | 80.0%  (59.8%-100.2%) | 85.0%  (69.4%-100.6%) | 48.0%  (28.4%-67.6%) | 64.4% | 35.6% |
| ALTO 8 | 32 | 73.3%  (57.5%-89.2%) | 33.3%  (9.5%-57.2%) | 68.75%  (52.7%-84.5%) | 38.5%  (12.0%-64.9%) | 60.0% | 40.0% |
| ALTO 9 | 32 | 73.3%  (57.5%-89.2%) | 33.3%  (9.5%-57.2%) | 68.75%  (52.7%-84.5%) | 38.5%  (12.0%-64.9%) | 60.0% | 40.0% |

**Abbreviation: ALTO=Alopecia Areata Assessment Tool**

# Supplementary Figure 1: ALTO Algorithms

The ALTO algorithms are scored according to the following rubric, modified from Li, et al^12^, and use the following questions:

1. Have you been diagnosed with alopecia areata by a dermatologist?
2. Have you been diagnosed with alopecia areata by a non-dermatologist health care provider (primary care physician, nurse practitioner, or physician assistant)?
3. Have you ever had round areas of hair loss on your face or scalp?
   1. If YES, did the hair ever grow back?
   2. If YES, did the hair loss last longer than 6 months?
4. Have you ever had complete loss of all the hair on your scalp?
5. Have you ever had complete loss of all the hair on your head AND body?


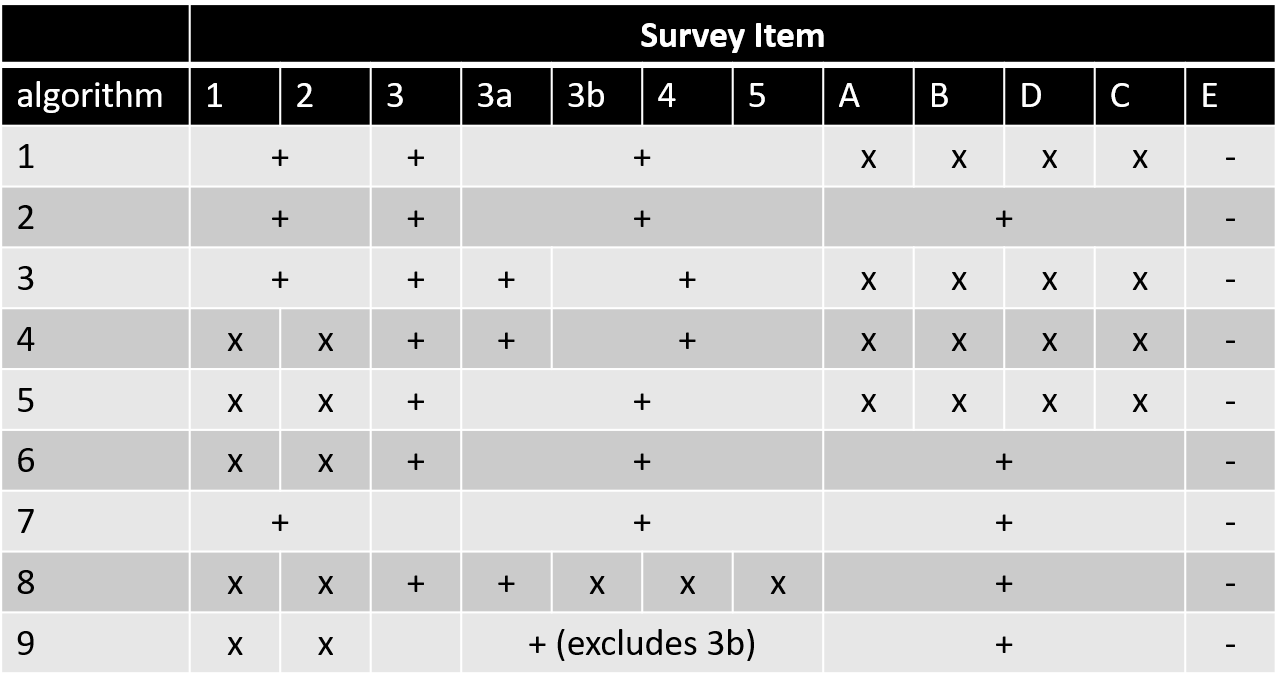
Respondents were also asked to select which (if any) of the images (A, B, C, or D) resemble their hair loss. A selection of “E” indicates that none of the images represent their hair loss.

Legend:

+: “yes” responses included in algorithm

-: “no” response included in algorithm

x: response not included in algorithm

Merged cells indicate that at least one option should be “yes”

For each algorithm, if all + are “yes” and – is “no”, the patient is assumed positive and otherwise negative.
